# The novel urinary proteomic classifier HF1 has similar diagnostic and prognostic utility to BNP in heart failure

**DOI:** 10.1002/ehf2.12708

**Published:** 2020-05-08

**Authors:** Ross T. Campbell, Adam Jasilek, Harald Mischak, Esther Nkuipou‐Kenfack, Agnieszka Latosinska, Paul I. Welsh, Colette E. Jackson, Jane Cannon, Alex McConnachie, Christian Delles, John J.V. McMurray

**Affiliations:** ^1^ Institute of Cardiovascular and Medical Sciences, BHF Glasgow Cardiovascular Research Centre University of Glasgow Glasgow G12 8TA UK; ^2^ Queen Elizabeth University Hospital Glasgow UK; ^3^ Roberson Centre for Biostatistics University of Glasgow Glasgow UK; ^4^ Mosaiques Diagnostics GmbH Hannover Germany; ^5^ Harefield Hospital Harefield London UK

**Keywords:** Heart failure, Biomarkers, Proteomics, Collagen, Diagnosis, Prognosis

## Abstract

**Aims:**

Measurement of B‐type natriuretic peptide (BNP) or N‐terminal pro‐BNP is recommended as part of the diagnostic workup of patients with suspected heart failure (HF). We evaluated the diagnostic and prognostic utility of the novel urinary proteomic classifier HF1, compared with BNP, in HF. HF1 consists of 85 unique urinary peptide fragments thought, mainly, to reflect collagen turnover.

**Methods and results:**

We performed urinary proteome analysis using capillary electrophoresis coupled with mass spectrometry in 829 participants. Of these, 622 had HF (504 had chronic HF and 118 acute HF) and 207 were controls (62 coronary heart disease patients without HF and 145 healthy controls). The area under the receiver operating characteristic (ROC) curve (AUC) using HF1 for the diagnosis of HF (cases vs. controls) was 0.94 (95% CI, 0.92–0.96). This compared with an AUC for BNP of 0.98 (95% CI, 0.97–0.99). Adding HF1 to BNP increased the AUC to 0.99 (0.98–0.99), *P* < 0.001, and led to a net reclassification improvement of 0.67 (95% CI, 0.54–0.77), *P* < 0.001. Among 433 HF patients followed up for a median of 989 days, we observed 186 deaths. HF1 had poorer predictive value to BNP for all‐cause mortality and did not add prognostic information when combined with BNP.

**Conclusions:**

The urinary proteomic classifier HF1 performed as well, diagnostically, as BNP and provided incremental diagnostic information when added to BNP. HF1 had less prognostic utility than BNP.

## Introduction

The diagnosis of heart failure (HF) is based on the presence of typical symptoms and signs, supported by investigative evidence. Measurement of a natriuretic peptide is the recommended first‐line investigation, with a normal plasma level suggesting an alternative diagnosis and an elevated level an indication for cardiac imaging.^1^ Demonstration of a structural or functional cardiac abnormality on imaging confirms the diagnosis, clarifies the cause of HF, and is essential for selection of the most appropriate therapy. Interpretation of natriuretic peptide levels can, however, be difficult in certain groups of patients, for example, those with atrial fibrillation, patients who are obese, individuals with renal impairment, and patients with pulmonary hypertension and right ventricular dysfunction secondary to chronic lung disease. Patients with HF with preserved ejection fraction (EF) (HFpEF) can be particularly difficult to diagnose, in part because they often have one or more of these co‐morbidities. Consequently, new biomarkers that might aid the diagnosis of HF are of great interest, especially if convenient for patients, if non‐invasive, and if cost‐effective and time effective.

The urinary proteome is a potential source of novel diagnostic biomarkers. Proteomic analysis using capillary electrophoresis coupled to mass spectrometry (CE‐MS) has previously been used to identify a panel of urinary peptides, termed HF1, which was able to distinguish patients with hypertension and diastolic impairment from healthy controls.^2^ The same classifier was able to identify those with diastolic dysfunction in a sample of 745 people randomly recruited from a Flemish population^3^ and distinguished patients with overt HF (16 hypertensive patients in New York Heart Association class II or III) from healthy controls (*n* = 16).^2^

Confirmation of these initial findings in a large cohort of patients with HF is still outstanding. Therefore, our aim was to test whether HF1 can discriminate clinical HF, both HF with reduced EF (HFrEF) and HFpEF from healthy controls and control subject with coronary heart disease (CHD). We also assessed how this classifier compared with B‐type natriuretic peptide (BNP), the current ‘gold standard’ first‐line test in the diagnostic pathway for HF, as well as the prognostic value of this marker.

## Methods

### Participants

Three participant cohorts were studied: (i) outpatients with chronic HF, (ii) patients hospitalized owing to HF, and (iii) controls (patients with CHD and healthy volunteers). Diagnosis of HF (both acute and chronic) was made using the European Society of Cardiology definition of HF recommended at the time of recruitment.^4^, ^5^ The chronic HF cohort included patients from two separate descriptive studies that enrolled participants between December 2006 and January 2009 and between March 2013 and December 2014.^6^, ^7^ Patients with acute HF were enrolled in a descriptive study between January 2013 and December 2014.^8^, ^9^ CHD and healthy controls were enrolled in a descriptive study between March 2013 and December 2014.^6^ The studies were each approved by the West of Scotland Research Ethics Committee, and each patient consented to measurement of potential biomarkers in their blood and urine.

All participants had measurement of plasma BNP and a detailed echocardiographic examination, including measurement of left ventricular EF using the Simpson biplane method.^10^ Potential participants were excluded from the healthy volunteer cohort if they had a BNP > 100 pg/mL, and patients with suspected acute HF were excluded if their BNP was <100 pg/mL at time of screening.

### Urinary proteomics analysis

#### Sample preparation and capillary electrophoresis coupled with mass spectrometry analysis

Urine samples were collected on the same day that echocardiograms and clinical assessment were performed and stored at −80°C. Proteomic analysis was undertaken by operators blind to participant group. CE‐MS analyses were performed using a P/ACE MDQ CE system (Beckman Coulter, Fullerton, CA) online coupled to a micro‐TOF MS (Bruker Daltonics, Bremen, Germany) as described previously.^11^, ^12^ Mass spectrometric data were evaluated as previously described,^13^ and the HF classifier, based on 85 urine peptides,^2^ was applied. The obtained peak lists characterize each polypeptide by its molecular mass, normalized CE migration time, and normalized signal intensity. HF1 proteomic classifier is a normalized, dimensionless variable; further details regarding the scoring of this proteomic classifier have previously been described.^13^

Further details of the urinary proteomic analysis are provided in the Supporting Information. Urine samples were collected on the same day as other study assessments, and no specific requirements were stipulated, for example, for an early‐morning sample or mid‐stream sample.

### Statistical analysis

Baseline characteristics are presented according to participant group. Continuous variables are described as median and inter‐quartile range, and categorical variables as counts and percentages. Boxplots are presented for the urinary classifier HF1 and split by the same groups.

Correlations of HF1 with log BNP, EF, and left ventricular size were calculated using Pearson's coefficient; and scatterplots are provided for each comparison. Receiver operating characteristic (ROC) was used to evaluate the diagnostic utility of HF1 as a continuous measure for discriminating between HF patients and controls, first as individual predictors and, then, as linear combinations, derived using logistic regression. *C*‐Statistics were calculated. The net reclassification improvement (NRI) was calculated to compare the predictive probabilities of models using BNP alone and BNP with HF1.

Patients who provided consent were followed up for vital status by record linkage through the Information Services Division of National Services Scotland. This record linkage has previously been described and provides near‐complete follow‐up with rare loss to follow‐up.^14^ For patients with follow‐up data, HF1 was grouped into tertiles, and Kaplan–Meier survival curves were generated for time to all‐cause death in relation to each tertile. *P*‐values for differences in curves were obtained using log rank tests. A Cox proportional hazard model was fitted using the Meta‐analysis Global Group in Chronic Heart Failure (MAGGIC) risk score as the only covariate.^15^ To assess the prognostic value of HF1, this model was extended to include this classifier as a continuous covariate. Hazard ratios, 95% confidence intervals (CIs), and *P*‐values are reported for each model.

All analyses were conducted using R® version 3.3.3.

## Results

We performed urinary proteome analysis by CE‐MS in 829 participants. Of these, 622 had HF (504 had chronic HF and 118 acute HF) and 207 were controls (62 with CHD and 145 healthy volunteers).

Baseline demographics of all three cohorts are shown in *Table* *1*. Patients with HF [median age 72 (66–80) years] were older than controls [66 (63–72) years]. Higher proportions of patients with HF and CHD controls were men, compared with the healthy volunteers. Renal function was better and haemoglobin was higher in healthy volunteers and CHD controls than in patients with HF. Liver function was similar in all cohorts.

**Table 1:** Baseline characteristics

	Heart failure					Controls		
		Acute HF		Chronic HF				
	All *n* = 622	HFrEF *n* = 79	HFpEF *n* = 39	HFrEF *n* = 430	HFpEF *n* = 74	All *n* = 184	CHD *n* = 45	Healthy *n* = 139
Age, years	72 [66–80]	75 [69–82]	77 [72–83]	71 [64–78]	74 [70–80]	65 [63–71]	65 [62–68]	66 [63–72]
Female sex	234 (38)	35 (44)	24 (62)	134 (31)	41 (55)	100 (54)	14 (31)	86 (62)
SBP, mmHg	132 [116–149]	132 [113–152]	135 [120–158]	131 [116–148]	135 [122–147]	140 [130–156]	138 [130–150]	140 [130–156]
NYHA class								
I	56 (9)	0 (0)	0 (0)	53 (12)	3 (4)	207 (100)	45 (100)	139 (100)
II	350 (56)	30 (38)	14 (36)	253 (59)	53 (72)	0 (0)	0 (0)	0 (0)
III	193 (31)	33 (42)	20 (51)	122 (28)	18 (24)	0 (0)	0 (0)	0 (0)
IV	23 (4)	16 (20)	5 (13)	2 (1)	0 (0)	0 (0)	0 (0)	0 (0)
Medical history								
Previous HF diagnosis	377 (61)	47 (60)	25 (64)	292 (68)	24 (32)	0 (0)	0 (0)	0 (0)
Hypertension	439 (71)	54 (68)	26 (67)	302 (70)	57 (77)	31 (17)	28 (62)	3 (2)
Myocardial infarction	282 (45)	35 (44)	15 (39)	209 (49)	23 (31)	39 (22)	39 (87)	0 (0)
Atrial fibrillation	300 (48)	38 (48)	20 (51)	199 (46)	43 (58)	0 (0)	0 (0)	0 (0)
TIA/CVA	91 (15)	14 (18)	7 (18)	52 (12)	18 (24)	0 (0)	0 (0)	0 (0)
Peripheral arterial disease	81 (13)	11 (14)	6 (15)	54 (13)	10 (14)	0 (0)	0 (0)	0 (0)
Diabetes	191 (31)	27 (34)	11 (28)	133 (31)	20 (27)	9 (6)	8 (18)	1 (1)
COPD	142 (10)	18 (23)	10 (26)	90 (21)	24 (32)	4 (2)	4 (9)	0 (0)
Medication								
ACEi	415 (67)	40 (51)^a^	17 (44)^a^	315 (73)	43 (58)	30 (16)	30 (67)	0 (0)
ARB	87 (14)	5 (6)^a^	9 (23)^a^	66 (15)	7 (10)	5 (3)	4 (9)	1 (1)
Beta‐blocker	441 (71)	45 (57)^a^	24 (62)^a^	326 (76)	46 (62)	37 (20)	37 (82)	0 (0)
MRA	129 (21)	6 (8)^a^	3 (8)^a^	116 (27)	4 (5)	1 (1)	1 (2)	0 (0)
Diuretic	546 (88)	48 (61)^a^	27 (69)^a^	400 (93)	71 (96)	7 (4)	7 (16)	0 (0)
Digoxin	115 (19)	1 (1)^a^	6 (15)^a^	83 (19)	25 (34)	0 (0)	0 (0)	0 (0)
Aspirin	311 (50)	37 (47)^a^	17 (44)^a^	220 (51)	37 (50)	43 (23)	43 (96)	0 (0)
Statin	431 (69)	46 (58)^a^	23 (59)^a^	310 (72)	52 (70)	44 (24)	43 (96)	1 (1)
Echocardiography								
EF, %	38 [30–45]	26 [22–36]	57 [53–66]	37 [30–41]	55 [51–58]	64 [60–68]	66 [62–70]	63 [60–68]
LVIDD, mm	56 [50–61]	59 [53–63]	51 [45–53]	57 [51–62]	48 [44–52]	48 [45–52]	48 [44–49]	48 [45–52]
Laboratory								
BNP, pg/mL	378 [175–795]	744 [442–1366]	464 [340–619]	328 [150–774]	222 [129–450]	28 [17–43]	46 [22–67]	25 [15–35]
Na^+^, mmol/L	139 [137–140]	137 [134–140]	138 [135–139]	139 [137–141]	139 [137–141]	139 [137–140]	138 [137–140]	139 [137–140]
Creatinine, μmol/L	103 [82–130]	100 [71–135]	97 [74–132]	105 [85–131]	99 [81–124]	70 [61–79]	73 [66–89]	67 [61–76]
eGFR, ml/min/1.73 m^2^	61 [44–76]	67 [44–83]	62 [38–76]	60 [45–77]	59 [48–71]	88 [76–99]	87 [75–102]	88 [80–98]
Hb, g/L	127 [115–140]	124 [111–139]	118 [105–133]	129 [116–143]	126 [113–134]	139 [132–149]	140 [132–149]	139 [132–149]
AST, mmol/L	21 [17–27]	24 [18–33]	22 [18–30]	21 [17–26]	20 [16–24]	21 [18–25]	23 [19–26]	21 [18–24]
ALT, mmol/L	18 [13–25]	18 [13–34]	21 [13–30]	18 [13–25]	17 [12–22]	21 [17–27]	22 [18–32]	20 [16–25]
Bilirubin, mmol/L	14 [8–16]	17 [11–26]	13 [10–21]	11 [8–15]	9 [7–13]	9 [8–13]	10 [7–13]	9 [8–13]
Proteomics								
HF1	1.35 [0.63–2.19]	1.92 [1.08–2.46]	1.31 [0.40–2.40]	1.30 [0.62–2.12]	1.16 [0.30–1.94]	−0.93 [−1.61 to −0.40]	−0.06 [−0.57–0.52]	−1.24 [−1.71 to −0.76]

Values are expressed as *n* (%) or median [inter‐quartile range]. HFrEF defined as ejection fraction < 50%.

ACEi, angiotensin converting enzyme inhibitor; ALT, alanine transaminase; ARB, angiotensin receptor agonist; AST, aspartate aminotransferase; BNP, B‐type natriuretic peptide; COPD, chronic obstructive pulmonary disease; CVA, cerebral vascular accident; EF, ejection fraction; eGFR, estimated glomerular filtration rate; Hb, haemoglobin; HF, heart failure; HFpEF, heart failure with preserved ejection fraction; HFrEF, heart failure with reduced ejection fraction; IHD, ischaemic heart disease; LVIDD, left ventricular internal diameter diastole; MRA, mineralocorticoid receptor antagonist; Na^+^, sodium; NYHA, New York Heart Association; SBP, systolic blood pressure; TIA, transient ischaemic attack.

a Medication pre‐admission.

Of the patients with chronic HF, 430 (85%) had HFrEF; of those with acute HF, the number with HFrEF was 79 (67%). There was a stepwise increase in BNP concentration from healthy controls (who had the lowest median BNP at 26 pg/mL) to patients with acute HFrEF (who had the highest median concentration at 744 pg/mL).

**Figure 1:**
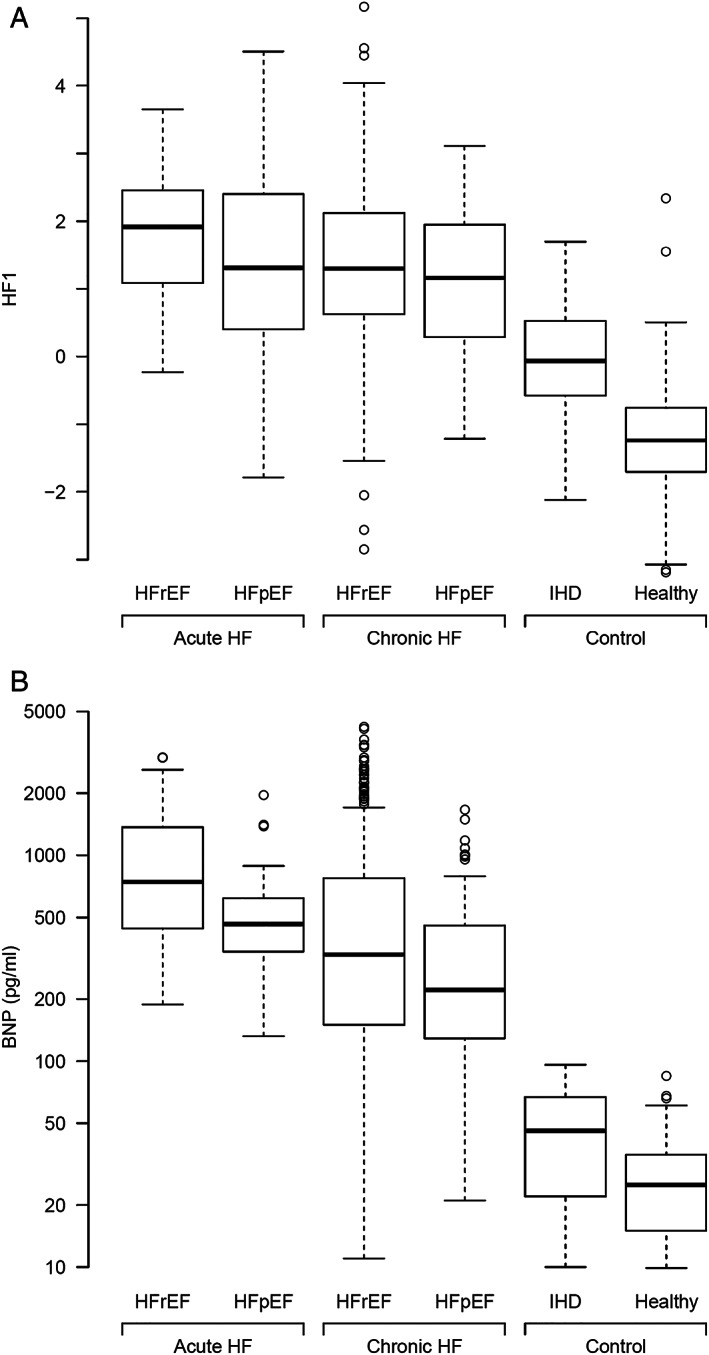
(A) Boxplots of HF1 urinary classifier by HF and control groups. HF1, heart failure 1 urinary proteomic classifier; HFpEF, heart failure with preserved ejection fraction; HFrEF, heart failure with reduced ejection fraction; IHD, ischaemic heart disease. (B) Boxplots of BNP by HF and control groups. BNP, B‐type natriuretic peptide; HFpEF, heart failure with preserved ejection fraction; HFrEF, heart failure with reduced ejection fraction; IHD, ischaemic heart disease.

**Figure 2:**
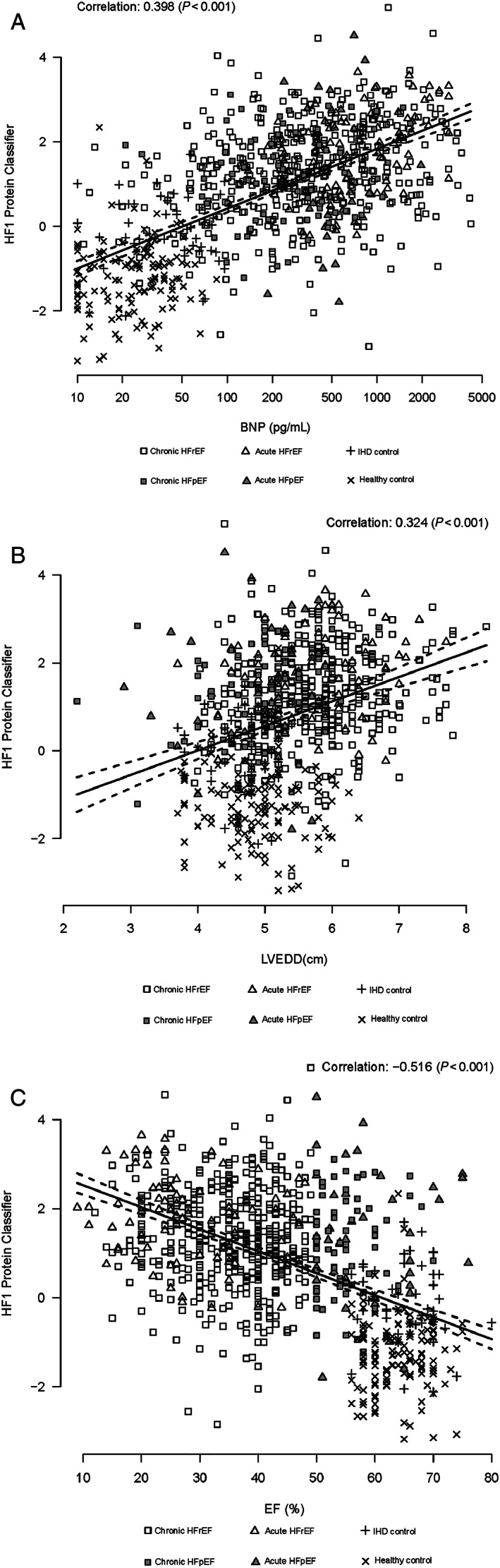
(A) Scatterplot HF1 and BNP. HF1, heart failure 1 urinary proteomic classifier; BNP, B‐type natriuretic peptide. Cases presented include acute and chronic HF and control subjects. (B) Scatterplot HF1 and LVEDD. HF1, heart failure 1 urinary proteomic classifier; LVEDD, left ventricular end‐diastolic dimension. Cases presented include acute and chronic HF, and control subjects. (C) Scatterplot HF1 and EF. HF1, heart failure 1 urinary proteomic classifier; EF, ejection fraction. Cases presented include acute and chronic HF and control subjects.

The results of urinary proteomic analysis and application of the HF1 classifier are shown in *Table* *1* and *Figure* *1*. Patients with HF had higher values for HF1 than had both the CHD controls and healthy volunteers. Patients with acute HF (both HFrEF and HFpEF) had the highest levels of HF1 (higher than those of patients with chronic HF). Scatterplots of HF1 and BNP, EF, and left ventricular size are shown in *Figure* *2*. There were positive correlations between HF1 and BNP (*r* = 0.40, *P* < 0.001) and ventricular size, as measured by left ventricular end‐diastolic dimension (*r* = 0.32, *P* < 0.001) and an inverse correlation between HF1 and EF (*r* = −0.52, *P* < 0.001).

### Diagnostic utility of HF1

**Figure 3:**
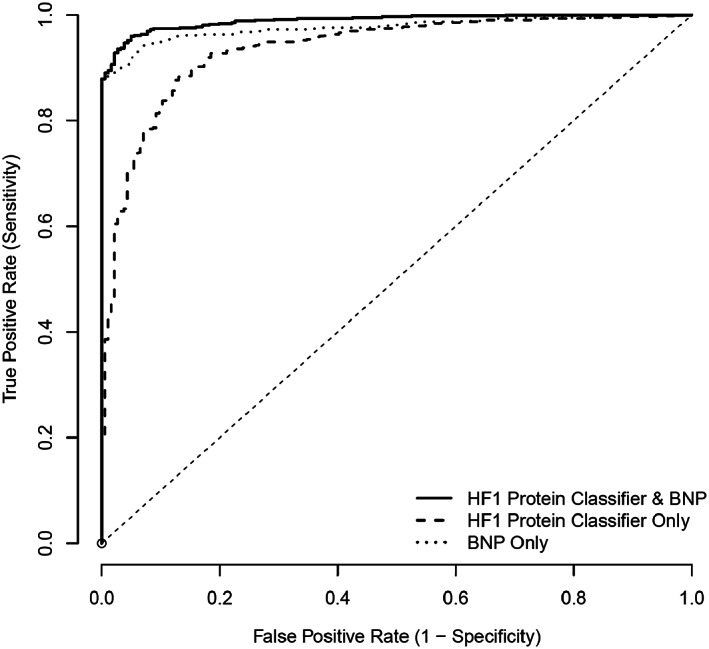
ROC for HF1 protein classifier, BNP, and HF1 + BNP: HF (both acute and chronic) vs. control (both CHD and healthy). BNP, B‐type natriuretic peptide; CHD, coronary heart disease; HF1, heart failure 1 urinary proteomic classifier; ROC, receiver operating characteristic.

The ROC curve for HF1, comparing definite cases of HF with control participants (both CHD controls and healthy volunteers), is shown in *Figure* *3*. The area under the ROC (AUC) for HF1 was 0.94 (95% CI, 0.92–0.96). This compared with an AUC for BNP of 0.98 (95% CI, 0.97–0.99). Adding HF1 to BNP resulted in AUC of 0.99 (95% CI, 0.98–0.99). The addition of HF1 to BNP resulted in an NRI of 0.67 (95% CI, 0.54–0.78), *P* < 0.001.

**Figure 4:**
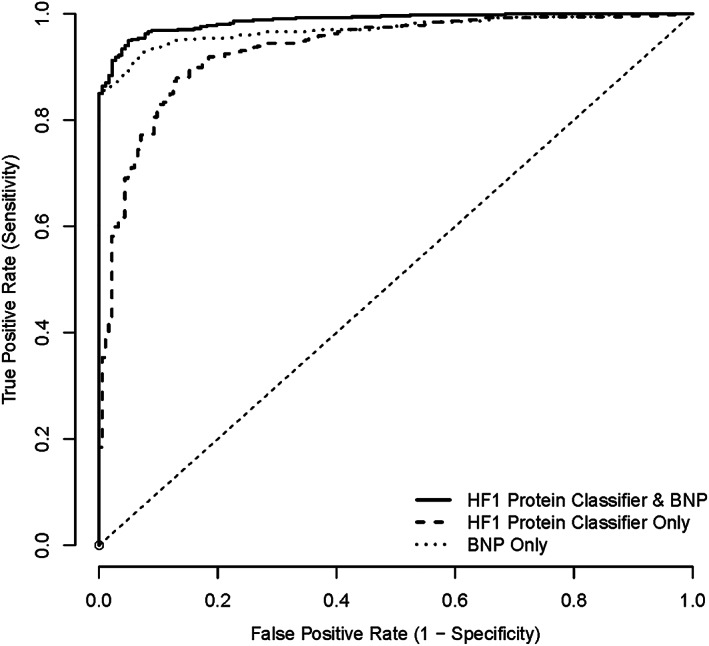
ROC for HF1 protein classifier, BNP, and HF1 + BNP: chronic HF vs. control (both CHD and healthy). BNP, B‐type natriuretic peptide; CHD, coronary heart disease; HF1, heart failure 1 urinary proteomic classifier; ROC, receiver operating characteristic.

When chronic HF cases (i.e. acute HF cases were excluded from analysis) were compared with controls only (*Figure* *4*), the AUC for HF1 was 0.93 (95% CI, 0.91–0.95); the corresponding AUC for BNP was 0.97 (95% CI, 0.96–0.98). Adding HF1 to BNP resulted in an AUC of 0.99 (0.98–0.99). The addition of HF1 to BNP resulted in an NRI of 0.71 (0.58–0.84), *P* < 0.001.

**Figure 5:**
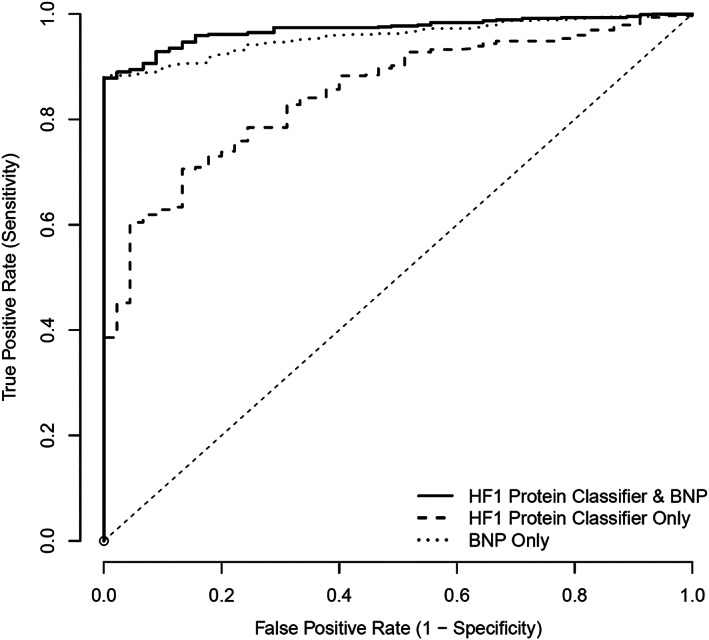
ROC for HF1 protein classifier, BNP, and HF1 + BNP: HF (both acute and chronic) vs. IHD control. BNP, B‐type natriuretic peptide; CHD, coronary heart disease; HF1, heart failure 1 urinary proteomic classifier; IHD, ischaemic heart disease; ROC, receiver operating characteristic.

When HF cases were compared with CHD controls only (*Figure* *5*), the AUC for HF1 was 0.85 (95% CI, 0.80–0.90); the corresponding AUC for BNP was 0.96 (95% CI, 0.94–0.97). Adding HF1 to BNP resulted in an AUC of 0.97 (0.96–0.98). The addition of HF1 to BNP resulted in an NRI of 0.68 (0.40–0.97), *P* < 0.001.

### Survival analysis

**Figure 6:**
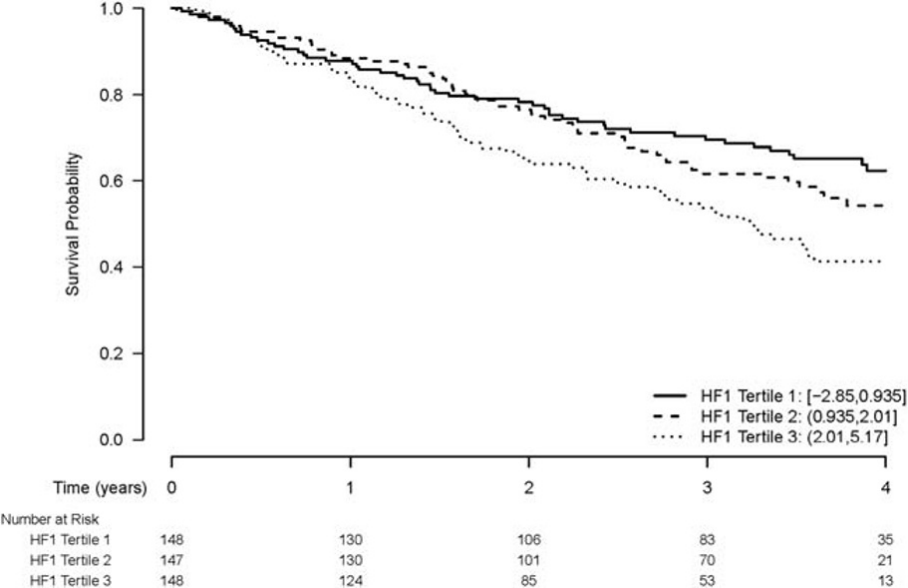
Kaplan–Meier estimates of time to death by tertile of HF1 protein classifier. Log Rank Test *P*‐value 0.004.

Of the 622 patients with HF, 433 patients provided consent to be followed up for vital status using long‐term record linkage. Follow‐up for vital status of the healthy and ischaemic heart disease controls was not available; and these subjects were therefore not included in the survival analyses. During a median follow‐up of 989 days, 186 patients died. Kaplan–Meier estimates of survival by tertile of HF1 are shown in *Figure* *6*, with a significant association between HF1 and mortality (*P* = 0.004). The additive value of HF1 to the MAGGIC prognostic risk score and to BNP is shown in *Table* *2*. HF1 did not retain prognostic predictive value in this cohort when added to either the MAGGIC risk score or BNP.

**Table 2:** All‐cause mortality in univariable and multivariable analyses

	*n*	HR (95% CI)	*P*‐value
Univariate analysis			
MAGGIC risk score^a^	440	1.10 (1.08–1.13)	<0.001
LogBNP^b^	443	1.53 (1.32–1.77)	<0.001
HF1^b^	443	1.22 (1.05–1.42)	<0.001
Multivariate analysis—MAGGIC + BNP			
MAGGIC risk score^a^	440	1.09 (1.06–1.12)	<0.001
LogBNP^b^	440	1.24 (1.06–1.45)	0.008
Multivariate analysis—MAGGIC + HF1			
MAGGIC risk score^a^	440	1.10 (1.07–1.13)	<0.001
HF1^b^	440	1.02 (0.89–1.17)	0.750
Multivariate analysis—BNP + HF1			
LogBNP^b^	443	1.49 (1.28–1.73)	<0.001
HF1^b^	443	1.07 (0.95–1.24)	0.211

*P*‐values derived from a Cox proportional hazards model.

CI, confidence interval; HR, hazard ratio; HF1, heart failure protein classifier 1; MAGGIC, Meta‐analysis Global Group in Chronic Heart Failure.

a Per unit increase.

b Per 1 standard deviation increase.

## Discussion

We describe the diagnostic and prognostic performance and potential utility of a novel urinary proteomic panel, HF1, in a large cohort of patents with HF, compared with healthy and CHD controls.

The first important observation from our study is the similarity in diagnostic utility of HF1 and BNP. The high AUC for BNP in our study is similar to that reported in previous studies assessing the diagnostic utility of BNP. In the Breathing Not Properly study, where 1586 patients who presented to an emergency department with acute dyspnoea had BNP analysed and a clinical diagnosis of HF was adjudicated by two cardiologists, BNP had an AUC of 0.91 (95% CI, 0.90–0.93).^16^ An elevated BNP of >100 pg/mL was the strongest independent predictor of having a diagnosis of HF, with an odds ratio of 30. Similarly, high AUCs have been reported for N‐terminal pro‐BNP (NT‐proBNP), for example, in the ProBNP Investigation of Dyspnea in the Emergency department study (PRIDE), where 600 patients presenting with acute dyspnoea had NT‐proBNP assessed.^17^ The AUC for NT‐proBNP in PRIDE was 0.94, and again elevated NT‐proBNP was the strongest predictor of a diagnosis of HF with an odds ratio of 44. The AUC in the present study was probably particularly high because we used BNP to include and exclude participants; that is, patients with HF had to have an elevated BNP, and normal controls a low BNP, to be eligible.^7^, ^8^ Because of this, HF1 was at a potential disadvantage, yet had a similar AUC to BNP and was additive when combined with BNP, with the combined AUC reaching 0.99.

The peptides in HF1 sequenced to date are not related to BNP and mainly reflect collagen fragments.^2^ This is supported by the moderate association between BNP and HF1, and the additive value of this proteomic panel to BNP. HF1 is therefore a marker of a different aspect of the disease process in HF. Where BNP is a compensatory up‐regulated mechanism, with BNP released in response to volume expansion and/or pressure overload resulting in increased wall stress, HF1 probably reflects changes in the extracellular matrix and, therefore, interstitial fibrosis.^2^ Although HF is a clinical syndrome, with a multitude of causes and different clinical phenotypes, myocardial interstitial fibrosis is a common pathophysiological process in most, if not all, of these.^18^ Future studies should build upon our findings by assessing the association between the proteomic classifier HF1 and direct and indirect markers of myocardial interstitial fibrosis such as endomyocardial biopsy and cardiac magnetic resonance (CMR) imaging.

Myocardial interstitial fibrosis is associated with worse clinical outcomes in patients with HFrEF and HFpEF.^19^, ^20^ Therefore, the proteomic marker HF1 should also have been associated with poorer prognosis in our cohort. This is what we found, although the relationship between HF1 and prognosis was not as strong as that with diagnosis. Although HF1 was an independent predictor of all‐cause mortality, it had a lower predictive value to BNP for all‐cause mortality and did not add prognostic information when combined with BNP. Neither BNP nor HF1 improved upon the prognostic information provided by the MAGGIC risk score. So our findings suggest that, in terms of prognosis, HF1 may be an alternative to BNP but has no added value. Further studies in larger cohorts are required to assess the predictive value of HF1 with more confidence.

### Study strengths and limitations

The strengths of our study were that the different cohorts of HF patients and controls were enrolled at a single investigative centre and each participant had a BNP level assayed and an echocardiographic measurement of EF using the Simpson biplane method. Both acute and chronic HF patients were included in our analysis as were patients with HFpEF and HFrEF. Limitations included that this was a retrospective analysis. Although a detailed echocardiogram was performed in each patient, only one measure of function (EF) and one measure of remodelling (left ventricular size) were included in the analysis. Further studies utilizing more advanced imaging techniques, such as myocardial strain or extracellular volume, using echocardiography and CMR, would provide further insights into the potential source of high HF1 in patients with HF. We studied only one clinical outcome, all‐cause death; and cardiac biomarkers may not predict all‐cause mortality as well as death due to cardiovascular causes. We did not have information of HF hospitalization, which is also predicted well by most cardiac biomarkers.

Although efforts were made to collect urine samples on the same day as other study assessments, this may not have been the case for every participant, although this was not recorded. There was no standard operating procedure for collection of urine; specifically, there were no requirements made for an early‐morning or mid‐stream sample. Although urinary peptides undergo changes throughout the day, by using a urinary proteomic classifier composed of multiple peptide (such as in our study), variance based on time of sample is reduced to non‐significant levels.^13^

## Conclusions

The urinary proteomic classifier HF1 is a novel biomarker that discriminates between patients with HF and both healthy controls and patients with CHD but no HF. Diagnostically, HF1 performed as well as BNP and provided incremental diagnostic information when added to BNP. HF1 also predicted all‐cause mortality but did not do this better than BNP or add to the prognostic value of BNP.

## Conflict of interest

H.M. is the founder and co‐owner of Mosaiques Diagnostics, which developed the CE‐MS technology for clinical application. E.N‐K. and A.L. are employed by Mosaiques Diagnostics.

## Funding

One cohort study was supported by funding from British Heart Foundation project grant, grant number PG/13/17/30050, and another by a Scottish Executive Chief Scientist Office project, grant number CZH/4/439. J.C. was supported by a fellowship from the British Society of Heart Failure. Funding to H.M. and E.N‐K. was provided by the European Commission via the HOMAGE project (HEALTH‐FP7‐305507). C.D. and J.J.V.M. are supported by a British Heart Foundation Centre of Research Excellence Award (reference numbers RE/13/5/30177 and RE/18/6/34217).

## Supporting information


**Table S1.** Univariate Cox model ‐ time to death: MAGGIC risk score.
**Table S2.** Univariate Cox model ‐ time to death: log (BNP).
**Table S3.** Univariate Cox model ‐ time to death: HF1.
**Table S4.** Multivariable Cox model ‐ time to death: MAGGIC risk score and log (BNP) continuous.
**Table S5.** Multivariable Cox model ‐ time to death: MAGGIC risk score and HF1 continuous.
**Table S6.** Multivariable Cox model ‐ time to death: log (BNP) and HF1 continuous.
