# Supplementary material for: The novel urinary proteomic classifier HF1 has similar diagnostic and prognostic utility to BNP in heart failure
Source: ESC Heart Fail. 2020 May 8;7(4):1595–604. doi: 10.1002/ehf2.12708 (PMC7373887; doi:10.1002/ehf2.12708)
Supplement: Supplementary file 1 — Table S1. Univariate Cox model ‐ time to death: MAGGIC risk score. Table S2. Univariate Cox model ‐ time to death: log (BNP). Table S3. Univariate Cox model ‐ time to death: HF1. Table S4. Multivariable Cox model ‐ time to death: MAGGIC risk score and log (BNP) continuous. Table S5. Multivariable Cox model ‐ time to death: MAGGIC risk score and HF1 continuous. Table S6. Multivariable Cox model ‐ time to death: log (BNP) and HF1 continuous. [file EHF2-7-1595-s001.pdf]

The novel urinary proteomic classifier HF1 has similar diagnostic and prognostic utility to B-type natriuretic peptide in heart failure.

Supplementary appendix

Additional survival analyses

Table 1: Univariate Cox model - time to death: MAGGIC risk score 3
Table 2: Univariate Cox model - time to death: log(BNP) 5
Table 3: Univariate Cox model - time to death: HF1 7
Table 4: Multivariable Cox model - time to death: MAGGIC risk score and log(BNP) continuous... 9
Table 5: Multivariable Cox model - time to death: MAGGIC risk score and HF1 continuous.. 11
Table 6: Multivariable Cox model - time to death: log(BNP) and HF1 continuous.. 13

Table 1: Univariate Cox model - time to death: MAGGIC risk score

Table 1.1: Univariate Cox model - time to death: MAGGIC risk score, truncated at 100 days

Variable	Co-efficient	Hazard Ratio	95% CI Hazard Ratio	P-value
MAGGIC risk score	0.036	1.037	(0.948, 1.135)	0.427
**Number of observations**	440			
**C-statistic**	0.568			

Table 1.2: Univariate Cox model - time to death: MAGGIC risk score, truncated at 200 days

Variable	Co-efficient	Hazard Ratio	95% CI Hazard Ratio	P-value
MAGGIC risk score	0.093	1.098	(1.039, 1.160)	<0.001
**Number of observations**	440			
**C-statistic**	0.668			

Table 1.3: Univariate Cox model - time to death: MAGGIC risk score, truncated at 300 days

Variable	Co-efficient	Hazard Ratio	95% CI Hazard Ratio	P-value
MAGGIC risk score	0.087	1.091	(1.043, 1.142)	<0.001
**Number of observations**	440			
**C-statistic**	0.663			

Table 1.4: Univariate Cox model - time to death: MAGGIC risk score, truncated at 500 days

Variable	Co-efficient	Hazard Ratio	95% CI Hazard Ratio	P-value
MAGGIC risk score	0.095	1.100	(1.060, 1.141)	<0.001
**Number of observations**	440			
**C-statistic**	0.669			

Table 1.5: Univariate Cox model - time to death: MAGGIC risk score, truncated at 750 days

Variable	Co-efficient	Hazard Ratio	95% CI Hazard Ratio	P-value
MAGGIC risk score	0.094	1.098	(1.067, 1.131)	<0.001
**Number of observations**	440			
**C-statistic**	0.665			

Table 1.6: Univariate Cox model - time to death: MAGGIC risk score, truncated at 1000 days

Variable	Co-efficient	Hazard Ratio	95% CI Hazard Ratio	P-value
MAGGIC risk score	0.106	1.112	(1.083, 1.143)	<0.001
**Number of observations**	440			
**C-statistic**	0.677			

Table 1.7: Univariate Cox model - time to death: MAGGIC risk score, not truncated

Variable	Co-efficient	Hazard Ratio	95% CI Hazard Ratio	P-value
MAGGIC risk score	0.098	1.103	(1.076, 1.131)	<0.001
**Number of observations**	440			
**C-statistic**	0.666			

Table 2: Univariate Cox model - time to death: log(BNP)

Table 2.1: Univariate Cox model - time to death: log(BNP), truncated at 100 days

Variable	Co-efficient	Hazard Ratio	95% CI Hazard Ratio	P-value
log(BNP), per SD	0.036	1.037	(0.948, 1.135)	0.427

**Number of observations**	440			
**C-statistic**	0.568			

Table 2.2: Univariate Cox model - time to death: log(BNP), truncated at 200 days

Variable	Co-efficient	Hazard Ratio	95% CI Hazard Ratio	P-value
log(BNP), per SD	0.093	1.098	(1.039, 1.160)	<0.001

**Number of observations**	440			
**C-statistic**	0.668			

Table 2.3: Univariate Cox model - time to death: log(BNP), truncated at 300 days

Variable	Co-efficient	Hazard Ratio	95% CI Hazard Ratio	P-value
log(BNP), per SD	0.087	1.091	(1.043, 1.142)	<0.001

**Number of observations**	440			
**C-statistic**	0.663			

Table 2.4: Univariate Cox model - time to death: log(BNP), truncated at 500 days

Variable	Co-efficient	Hazard Ratio	95% CI Hazard Ratio	P-value
log(BNP), per SD	0.095	1.100	(1.060, 1.141)	<0.001

**Number of observations**	440			
**C-statistic**	0.669			

Table 2.5: Univariate Cox model - time to death: log(BNP), truncated at 750 days

Variable	Co-efficient	Hazard Ratio	95% CI Hazard Ratio	P-value
log(BNP), per SD	0.094	1.098	(1.067, 1.131)	<0.001

**Number of observations**	440			
**C-statistic**	0.665			

Table 2.6: Univariate Cox model - time to death: log(BNP), truncated at 1000 days

Variable	Co-efficient	Hazard Ratio	95% CI Hazard Ratio	P-value
log(BNP), per SD	0.106	1.112	(1.083, 1.143)	<0.001

**Number of observations**	440			
**C-statistic**	0.677			

Table 2.7: Univariate Cox model - time to death: log(BNP), not truncated				
**Variable**	**Co-efficient**	**Hazard Ratio**	**95% CI Hazard Ratio**	**P-value**
log(BNP), per SD	0.098	1.103	(1.076, 1.131)	<0.001
**Number of observations**	440			
**C-statistic**	0.666			

Table 3: Univariate Cox model - time to death: HF1

Table 3.1: Univariate Cox model - time to death: HF1, truncated at 100 days

Variable	Co-efficient	Hazard Ratio	95% CI Hazard Ratio	P-value
HF1, per SD	-0.138	0.871	(0.499, 1.523)	0.629
**Number of observations**	443			
**C-statistic**	0.536			

Table 3.2: Univariate Cox model - time to death: HF1, truncated at 200 days

Variable	Co-efficient	Hazard Ratio	95% CI Hazard Ratio	P-value
HF1, per SD	0.036	1.036	(0.740, 1.452)	0.835
**Number of observations**	443			
**C-statistic**	0.515			

Table 3.3: Univariate Cox model - time to death: HF1, truncated at 300 days

Variable	Co-efficient	Hazard Ratio	95% CI Hazard Ratio	P-value
HF1, per SD	0.102	1.108	(0.838, 1.466)	0.473
**Number of observations**	443			
**C-statistic**	0.531			

Table 3.4: Univariate Cox model - time to death: HF1, truncated at 500 days

Variable	Co-efficient	Hazard Ratio	95% CI Hazard Ratio	P-value
HF1, per SD	0.149	1.161	(0.927, 1.455)	0.194
**Number of observations**	443			
**C-statistic**	0.551			

Table 3.5: Univariate Cox model - time to death: HF1, truncated at 750 days

Variable	Co-efficient	Hazard Ratio	95% CI Hazard Ratio	P-value
HF1, per SD	0.206	1.228	(1.024, 1.473)	0.027
**Number of observations**	443			
**C-statistic**	0.561			

Table 3.6: Univariate Cox model - time to death: HF1, truncated at 1000 days

Variable	Co-efficient	Hazard Ratio	95% CI Hazard Ratio	P-value
HF1, per SD	0.199	1.220	(1.035, 1.439)	0.018
**Number of observations**	443			
**C-statistic**	0.558			

Table 3.7: Univariate Cox model - time to death: HF1, not truncated				
**Variable**	**Co-efficient**	**Hazard Ratio**	**95% CI Hazard Ratio**	**P-value**
HF1, per SD	0.200	1.222	(1.054, 1.416)	0.008
**Number of observations**	443			
**C-statistic**	0.563			

Table 4: Multivariable Cox model - time to death: MAGGIC risk score and log(BNP) continuous

Table 4.1: Multivariable Cox model - time to death: MAGGIC risk score and log(BNP) continuous, truncated at 100 days

Variable	Co-efficient	Hazard Ratio	95% CI Hazard Ratio	P-value
MAGGIC risk score	0.007	1.007	(0.911, 1.113)	0.891
log(BNP), per SD	0.382	1.465	(0.948, 2.264)	0.086
**Number of observations**	440			
**C-statistic**	0.679			

Table 4.2: Multivariable Cox model - time to death: MAGGIC risk score and log(BNP) continuous, truncated at 200 days

Variable	Co-efficient	Hazard Ratio	95% CI Hazard Ratio	P-value
MAGGIC risk score	0.076	1.079	(1.017, 1.145)	0.012
log(BNP), per SD	0.256	1.292	(0.991, 1.685)	0.059
**Number of observations**	440			
**C-statistic**	0.683			

Table 4.3: Multivariable Cox model - time to death: MAGGIC risk score and log(BNP) continuous, truncated at 300 days

Variable	Co-efficient	Hazard Ratio	95% CI Hazard Ratio	P-value
MAGGIC risk score	0.076	1.079	(1.028, 1.133)	0.002
log(BNP), per SD	0.180	1.197	(0.947, 1.514)	0.132
**Number of observations**	440			
**C-statistic**	0.668			

Table 4.4: Multivariable Cox model - time to death: MAGGIC risk score and log(BNP) continuous, truncated at 500 days

Variable	Co-efficient	Hazard Ratio	95% CI Hazard Ratio	P-value
MAGGIC risk score	0.087	1.091	(1.050, 1.135)	<0.001
log(BNP), per SD	0.129	1.138	(0.937, 1.382)	0.191
**Number of observations**	440			
**C-statistic**	0.672			

Table 4.5: Multivariable Cox model - time to death: MAGGIC risk score and log(BNP) continuous, truncated at 750 days

Variable	Co-efficient	Hazard Ratio	95% CI Hazard Ratio	P-value
MAGGIC risk score	0.085	1.089	(1.055, 1.123)	<0.001
log(BNP), per SD	0.150	1.162	(0.995, 1.357)	0.058
**Number of observations**	440			
**C-statistic**	0.671			

Table 4.6: Multivariable Cox model - time to death: MAGGIC risk score and log(BNP) continuous, truncated at 1000 days

Co-efficient	Hazard Ratio	95% CI Hazard Ratio	P-value	
MAGGIC risk score	0.099	1.104	(1.073, 1.136)	<0.001
log(BNP), per SD	0.131	1.139	(0.990, 1.311)	0.069
**Number of observations**	440			
**C-statistic**	0.684			

Table 4.7: Multivariable Cox model - time to death: MAGGIC risk score and log(BNP) continuous, not truncated

Variable	Co-efficient	Hazard Ratio	95% CI Hazard Ratio	P-value
MAGGIC risk score	0.090	1.095	(1.066, 1.124)	<0.001
log(BNP), per SD	0.128	1.137	(0.999, 1.294)	0.052
**Number of observations**	440			
**C-statistic**	0.674			

Table 5: Multivariable Cox model - time to death: MAGGIC risk score and HF1 continuous

Table 5.1: Multivariable Cox model - time to death: MAGGIC risk score and HF1 continuous, truncated at 100 days

Variable	Co-efficient	Hazard Ratio	95% CI Hazard Ratio	P-value
MAGGIC risk score	0.047	1.048	(0.953, 1.153)	0.333
HF1, per SD	-0.223	0.800	(0.450, 1.424)	0.449
**Number of observations**	440			
**C-statistic**	0.586			

Table 5.2: Multivariable Cox model - time to death: MAGGIC risk score and HF1 continuous, truncated at 200 days

Variable	Co-efficient	Hazard Ratio	95% CI Hazard Ratio	P-value
MAGGIC risk score	0.102	1.107	(1.044, 1.174)	<0.001
HF1, per SD	-0.159	0.853	(0.600, 1.211)	0.374
**Number of observations**	440			
**C-statistic**	0.671			

Table 5.3: Multivariable Cox model - time to death: MAGGIC risk score and HF1 continuous, truncated at 300 days

Variable	Co-efficient	Hazard Ratio	95% CI Hazard Ratio	P-value
MAGGIC risk score	0.091	1.096	(1.044, 1.150)	<0.001
HF1, per SD	-0.075	0.928	(0.693, 1.243)	0.615
**Number of observations**	440			
**C-statistic**	0.663			

Table 5.4: Multivariable Cox model - time to death: MAGGIC risk score and HF1 continuous, truncated at 500 days

Variable	Co-efficient	Hazard Ratio	95% CI Hazard Ratio	P-value
MAGGIC risk score	0.097	1.102	(1.060, 1.145)	<0.001
HF1, per SD	-0.038	0.963	(0.761, 1.219)	0.753
**Number of observations**	440			
**C-statistic**	0.671			

Table 5.5: Multivariable Cox model - time to death: MAGGIC risk score and HF1 continuous, truncated at 750 days

Variable	Co-efficient	Hazard Ratio	95% CI Hazard Ratio	P-value
MAGGIC risk score	0.093	1.097	(1.063, 1.131)	<0.001
HF1, per SD	0.026	1.026	(0.848, 1.243)	0.791
**Number of observations**	440			
**C-statistic**	0.666			

Table 5.6: Multivariable Cox model - time to death: MAGGIC risk score and HF1 continuous, truncated at 1000 days

Variable	Co-efficient	Hazard Ratio	95% CI Hazard Ratio	P-value
MAGGIC risk score	0.107	1.113	(1.081, 1.145)	<0.001
HF1, per SD	-0.006	0.994	(0.835, 1.182)	0.945
**Number of observations**	440			
**C-statistic**	0.677			

Table 5.7: Multivariable Cox model - time to death: MAGGIC risk score and HF1 continuous, not truncated

Variable	Co-efficient	Hazard Ratio	95% CI Hazard Ratio	P-value
MAGGIC risk score	0.097	1.102	(1.074, 1.131)	<0.001
HF1, per SD	0.025	1.025	(0.878, 1.197)	0.750
**Number of observations**	440			
**C-statistic**	0.667			

Table 6: Multivariable Cox model - time to death: log(BNP) and HF1 continuous

Table 6.1: Multivariable Cox model - time to death: log(BNP) and HF1 continuous, truncated at 100 days

Variable	Co-efficient	Hazard Ratio	95% CI Hazard Ratio	P-value
log(BNP), per SD	0.790	2.204	(1.175, 4.134)	0.014
HF1, per SD	-0.301	0.740	(0.427, 1.282)	0.283
**Number of observations**	443			
**C-statistic**	0.717			

Table 6.2: Multivariable Cox model - time to death: log(BNP) and HF1 continuous, truncated at 200 days

Variable	Co-efficient	Hazard Ratio	95% CI Hazard Ratio	P-value
log(BNP), per SD	0.598	1.818	(1.256, 2.633)	0.002
HF1, per SD	-0.109	0.897	(0.639, 1.258)	0.528
**Number of observations**	443			
**C-statistic**	0.649			

Table 6.3: Multivariable Cox model - time to death: log(BNP) and HF1 continuous, truncated at 300 days

Variable	Co-efficient	Hazard Ratio	95% CI Hazard Ratio	P-value
log(BNP), per SD	0.452	1.572	(1.163, 2.125)	0.003
HF1, per SD	-0.013	0.987	(0.742, 1.311)	0.926
**Number of observations**	443			
**C-statistic**	0.620			

Table 6.4: Multivariable Cox model - time to death: log(BNP) and HF1 continuous, truncated at 500 days

Variable	Co-efficient	Hazard Ratio	95% CI Hazard Ratio	P-value
log(BNP), per SD	0.431	1.538	(1.209, 1.957)	<0.001
HF1, per SD	0.037	1.037	(0.825, 1.305)	0.754
**Number of observations**	443			
**C-statistic**	0.622			

Table 6.5: Multivariable Cox model - time to death: log(BNP) and HF1 continuous, truncated at 750 days

Variable	Co-efficient	Hazard Ratio	95% CI Hazard Ratio	P-value
log(BNP), per SD	0.418	1.518	(1.253, 1.840)	<0.001
HF1, per SD	0.093	1.097	(0.911, 1.321)	0.330
**Number of observations**	443			
**C-statistic**	0.623			

Table 6.6: Multivariable Cox model - time to death: log(BNP) and HF1 continuous, truncated at 1000 days

Variable	Co-efficient	Hazard Ratio	95% CI Hazard Ratio	P-value
log(BNP), per SD	0.436	1.547	(1.302, 1.838)	<0.001
HF1, per SD	0.081	1.084	(0.916, 1.283)	0.347
**Number of observations**	443			
**C-statistic**	0.628			

Table 6.7: Multivariable Cox model - time to death: log(BNP) and HF1 continuous, not truncated

Variable	Co-efficient	Hazard Ratio	95% CI Hazard Ratio	P-value
log(BNP), per SD	0.399	1.491	(1.281, 1.734)	<0.001
HF1, per SD	0.095	1.100	(0.947, 1.278)	0.211
**Number of observations**	443			
**C-statistic**	0.626			
